# Beyond Necrotizing Enterocolitis: Other Clinical Advantages of an Exclusive Human Milk Diet

**DOI:** 10.1089/bfm.2017.0192

**Published:** 2018-07-01

**Authors:** Amy B. Hair, David J. Rechtman, Martin L. Lee, Victoria Niklas

**Affiliations:** ^1^ Section of Neonatology, Department of Pediatrics, Baylor College of Medicine, Texas Children's Hospital, Houston, Texas.; ^2^ Prolacta Bioscience, Duarte, California.; ^3^ Fielding School of Public Health, University California Los Angeles, Los Angeles, California.; ^4^ Department of Pediatrics, David Geffen School of Medicine at University California Los Angeles, Los Angeles, California.

**Keywords:** exclusive human milk diet (EHMD), extremely premature, oxygen therapy, ventilator support

## Abstract

***Objective:*** Articles previously published by Sullivan et al. and Cristofalo et al. were reanalyzed using the proportion of cow milk-based nutrition received to determine whether that affected clinical outcomes during hospitalization for infants birth weight 500–1250 g. Abrams et al. showed in the same cohort incidences of necrotizing enterocolitis (NEC), NEC requiring surgery and sepsis increased proportionally to the amount of dietary cow milk.

***Methods:*** The data from the two studies conducted under essentially the same protocol were combined yielding a cohort of 260 infants receiving a diet ranging from 0% to 100% cow milk. Data analysis utilized negative binomial regression which mitigates differences between subjects in terms of their time on study by incorporating that number into the statistical model. The percent of cow milk-based nutrition was the only predictor investigated.

***Results:*** For all outcomes the larger the amount of cow's milk in the diet the greater the number of days of that intervention required. A trend toward statistical significance was seen for ventilator days; however, only parenteral nutrition (PN) days and days to full feeds achieved statistical significance.

***Conclusions:*** Incorporation of any cow milk-based nutrition into the diet of extremely premature infants correlates with more days on PN and a longer time to achieve full feeds. There was a nonstatistically significant trend toward increased ventilator days. These represent additional clinical consequences of the use of any cow milk-based protein in feeding EP infants.

## Introduction

Human milk (HM) is the ideal source of nutrition for all infants, including those born prematurely. Its use can provide benefits related to host defense, reduced infection rate, gastrointestinal maturation, improved neurodevelopmental outcomes, and prevention of long-term metabolic and cardiovascular disease.^1^ As such, the American Academy of Pediatrics^2^ recommends that mother's own milk or donor HM should be used as the foundation for enteral nutrition in preterm, very low birth weight (VLBW) infants. However, the caloric requirements required to maintain the fetal growth trajectory after birth in VLBW infants are not met with native breast milk and require fortification from an external source to meet the nutritional needs of preterm infants.^3,4^

We have previously shown that the average energy content in a nationwide sample of donor HM was 19 kcal/oz with 25% of samples falling below 17.3 kcal/oz and 65% of the samples below 20 kcal/oz.^3^ Moreover, a similar analysis of both donor and mother's own milk demonstrated that most breast milk is sufficiently below the recommended nutritional content required to maintain adequate growth in preterm infants. Nearly 80% of samples had a fat content <4 g/dL, while 56% had a protein content <1.5 g/dL, and 67% had an energy density <67 kcal/dL,^5^ well below recommended standards for this group of infants.

Two randomized clinical trials have shown that an exclusive HM diet (EHMD) significantly decreases the rates of necrotizing enterocolitis (NEC), sepsis, days of parenteral nutrition (PN), and death.^6–8^ A number of other important clinical end points were evaluated in those studies as secondary outcomes. It is the purpose of this article to report on a post hoc analysis of those findings in the complete cohort of study subjects from those previously published studies.

## Materials and Methods

An EHMD was evaluated in two clinical trials of premature infants born at <1,250 g birth weight.^6,7^ Both trials used the same approach to feeding and evaluation of outcomes. The first of these trials involved infants whose mothers had committed to providing breast milk during the neonatal intensive care unit stay. For these studies, infants were randomly assigned to either an EHMD comprising of mother's own milk or donor HM and fortification with HM-based HM fortifier (Prolacta Bioscience, City of Industry, CA) or to a routine approach in which mother's own milk was fortified with a cow milk-based fortifier. In that group, if mother's own milk was not available, a cow milk-based infant formula product was used to supplement the mother's own milk, but donor HM was not provided, as this did not represent standard of care at the time the trial was conducted.

The second trial included infants whose mothers, for whatever reason, were unable or unwilling to provide breast milk during the neonatal intensive care unit stay. In that trial, infants were assigned randomly to receive either an all HM-based diet using donor HM and HM-based HM fortifier (Prolact+) or cow milk-based formula products. Otherwise, the protocol was essentially the same as for the first trial. Together, the two trials enrolled a total of 260 extremely premature infants.

In addition to the results previously reported, the protocol called for collecting data on PN days, central line days, ventilator days, days of oxygen supplementation, presence of patent ductus arteriosus and therapy (if any), and days until full enteral feeds were achieved. These results were evaluated for the combined cohort of infants in both studies.

### Statistical analysis

A negative binomial regression model was used to examine the effect of varying amounts of cow milk-based products as a function of these outcomes.^9^ Primary analytical end points are count data. However, the negative binomial approach was chosen over a Poisson model because the “risk” of an additional day of a given outcome could very easily vary from infant to infant.

In all statistical evaluations, significance was defined as a *p*-value less than 0.05.

## Results

The characteristics of the infants enrolled in both the Sullivan and the Cristofalo studies were similar between the groups receiving EHMD and those receiving the control diet. These data are shown in Table 1.

**Table 1.** Demographic and Baseline Medical Characteristics of the Participants in the Combined Sullivan and Cristofalo Studies

*Parameter*	*HUM results (*n* = 167)*	*BOV results (*n* = 93)*	p
BW (g), mean ± SD	939 ± 192	938 ± 200	0.996
GA (weeks), mean ± SD	27.3 ± 2.1	27.3 ± 2.1	0.83
Male/female, *n* (%)	69/98 (41/59)	47/46 (51/49)	0.15
SGA, *n* (%)	15 (9)	10 (11)	0.64
APGAR <6, *n* (%)	13 (8)	11 (12)	0.28
Black race, *n* (%)	50 (30)	21 (23)	0.20
Antenatal steroids, *n* (%)	128 (77)	71 (76)	0.83
Mech vent at entry, *n* (%)	105 (63)	53 (57)	0.95

HUM, human milk arm; BOV, bovine milk arm.

The results of the secondary analysis are shown below in Table 2.

**Table 2.** Endpoints Considered in This Study

	*EHM*	*Control*	p^*^
Parenteral nutrition	24.2 ± 15.6	25.9 ± 16.7	0.028
Central line use	24.4 ± 18.3	25.4 ± 18.9	0.23
Ventilator support	24.6 ± 24.5 (median = 16)	29.3 ± 28.0 (median = 21)	0.06
Oxygen therapy	33.7 ± 31.1 (median = 26)	35.9 ± 30.4 (median = 28)	0.90
Time to full feeds	25.3 ± 15.0	26.2 ± 14.3	0.0028

^*^ Significance of a % cow's milk diet based on negative binomial regression model.

EHM, exclusive human milk.

Infants fed an EHMD had clinically and statistically significantly fewer days on PN and attained full feeds significantly sooner than the control group. The EHM group also showed a trend toward fewer ventilator days, but this did not reach statistical significance.

**FIG. 1.**
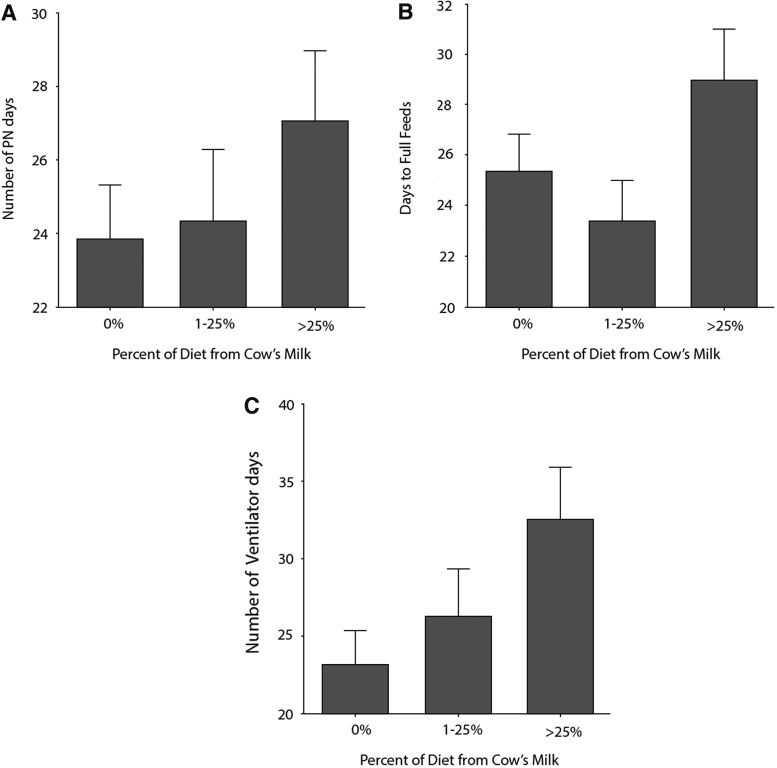
The percentage of diet from cow's milk influences clinical outcomes in infants fed human milk. **(A)** Number of parenteral nutrition (PN) days (*p* = 0.028), **(B)** days to full feeds (*p* = 0.0028), and **(C)** number of days on ventilator (*p* = 0.06).

It is interesting to note that there appears to be a dose–response effect for these end points based on the amount of cow milk protein as a proportion of the infants' diet. This is particularly pronounced when the days on PN and days requiring ventilator assistance are considered as seen in Figure 1.

## Discussion

The need for PN was significantly related to the amount of cow's milk in the infant's diet. This comports nicely with a reanalysis of the data from the Sullivan trial that was published by Ghandehari et al.,^10^ which demonstrated that the EHM group had a 11–14% lower likelihood of being on PN at any given day of the study. Since PN is tapered as enteral feeds increase, it is unsurprising that the lower requirement for PN was accompanied by more rapid attainment of full enteral feeds. The dose–response nature of the effect would seem to support the argument that cow milk components are not merely associated with but causative of the increased need for PN and delayed attainment of full feeds in the control group. Moreover, as PN carries significant costs, lowering those costs through a reduced need for PN may result in substantial savings to the institution.

It is interesting to note that despite the fewer days of PN required and the more rapid attainment of full enteral feeds as a function of the reduction in the amount of cow's milk in the diet, no significant association with the amount of exposure to cow's milk could be demonstrated for days of central line placement. This would seem to imply that central lines were kept in for reasons other than delivery of PN. Moreover, we have previously shown that the risk of sepsis was directly proportional to the amount of cow milk protein in the diet.^8^ Therefore, it would seem that the increased risk with regard to sepsis in the control group cannot be explained by prolonged presence of a central line and may be due to a direct effect of the cow's milk-based elements in the diet. Sepsis is an expensive complication and reducing its incidence could also result in substantial savings for the institution.

Finally, the trend seen toward fewer ventilator days as a function of less cow's milk in the diet is consistent with the findings reported in a study^11^ of a HM cream based supplement to the EHMD. This study showed significantly earlier discharge of extremely premature infants at lower corrected gestational ages with use of the cream supplement as opposed to the EHMD without it. The argument can be made that increasing the amount of HM, and particularly the amount of HM fat, helps to improve pulmonary outcomes. Thus, the infants receiving the EHMD in these studies did better than those who received less or no breast milk while those who received supplementary cream in the later study did even better.

Limitations of this analysis include the fact that it is a secondary analysis combining the results of two previously reported clinical trials where the end points did not show a difference between the groups. However, this changed when the binary study group variable was converted into the amount of exposure to cow's milk. This suggests that the effect of cow's milk in the premature infant's diet is really dose dependent rather than necessarily an all-or-none response, as previously described by Meinzen-Derr et al.^12^ Given the fact that upon combining the studies the difference is seen may indicate this to simply be a matter of insufficient power in each of the original trials to detect these differences.

For the trials involved in this analysis, premature infant formula rather than donor milk was used routinely to supplement shortfalls in mother's own milk in the control arm. As already noted, the trial was designed as an intervention compared with what was, at the time, standard of care. Currently less than half the level III or higher neonatal intensive care units in the United States use any donor milk and a smaller proportion use it routinely^13^; thus, donor milk is still not the de facto standard of care regardless of the exhortations from various professional groups. Moreover, as previously reported,^8^ the feeding practices in the trials allowed us to examine the effect of the amount of cow milk protein in the diet on the various study end points, including the outcomes examined in the current evaluation.

The results of this analysis indicate that there are benefits of using the EHMD both to the patient in terms of outcomes and potentially to the institution in terms of costs over and above those from the previously reported reductions in the risk of NEC in general, surgery for NEC, sepsis, and mortality.
